# Blunt cardiac rupture of left ventricular apex without external chest injury in a young man: a case report

**DOI:** 10.1093/fsr/owaf044

**Published:** 2025-11-14

**Authors:** Qing Gao, Huine Liu, Xiaoshan Tan, Weisheng Huang, Meichen Pan, Yiwu Zhou, Hongmei Dong

**Affiliations:** Department of Forensic Medicine, Tongji Medical College, Huazhong University of Science and Technology, Wuhan, China; Taikang Medical School (School of Basic Medical Sciences), Wuhan University, Wuhan, China; Department of Forensic Medicine, Tongji Medical College, Huazhong University of Science and Technology, Wuhan, China; Department of Forensic Medicine, Tongji Medical College, Huazhong University of Science and Technology, Wuhan, China; Department of Forensic Medicine, Tongji Medical College, Huazhong University of Science and Technology, Wuhan, China; Department of Forensic Medicine, Tongji Medical College, Huazhong University of Science and Technology, Wuhan, China; Department of Forensic Medicine, Tongji Medical College, Huazhong University of Science and Technology, Wuhan, China; Department of Forensic Medicine, Tongji Medical College, Huazhong University of Science and Technology, Wuhan, China

**Keywords:** forensic sciences, forensic pathology, cardiac rupture, myocardial infarction, blunt cardiac trauma, cardiopulmonary resuscitation, Takotsubo cardiomyopathy, cause of death

## Abstract

Cardiac rupture in blunt cardiac injuries, though uncommon, can be fatal. A particularly rare occurrence is blunt cardiac rupture of the left ventricle without external chest injury. Herein, we report a case of cardiac rupture without external chest injuries in a 21-year-old man who died after a physical altercation. The cause of death was attributed to cardiac rupture caused by blunt force. We analysed the differential diagnoses of cardiac rupture with intact pericardium, including cardiac diseases, blunt chest injury, cardiopulmonary resuscitation, and Takotsubo cardiomyopathy. Additionally, we discussed the relevant factors for cardiac rupture in this case, including sudden enormous force, cardiac cycle, chest wall compliance, and alcohol consumption. Furthermore, we highlighted that no explicable cause other than cardiac rupture accounted for the death.

## Introduction

Cardiac rupture is an uncommon but fatal blunt cardiac injury [1]. Blunt cardiac injuries are commonly caused by traffic accidents and falling from significant heights and are usually accompanied by external and internal chest injuries such as bruises, rib fractures, sternum fractures, pulmonary contusions, and bronchial injuries [2, 3]. Some reports indicate that cardiac ruptures can occur in various parts of the heart, most frequently in the right atrium, followed by the right ventricle and the left atrium [1, 4, 5]. However, left ventricular rupture is unusual. Thus, blunt cardiac rupture of the left ventricle occurring in a healthy heart with no external chest injuries is exceedingly rare [6]. Herein, we present the case of a 21-year-old man who sustained cardiac rupture with an intact pericardium and no other apparent external chest injuries during a physical confrontation.

## Case presentation

This study was approved by the relevant judicial authorities. The requirement for ethical review was waived by the Ethics Committee of Tongji Medical College, Huazhong University of Science and Technology. Written informed consent was obtained from the victim’s family for both the study and the publication of this case report. The victim, a slender 21-year-old man, had a physical confrontation with his friend (the perpetrator, a stronger male adult due to long-term agricultural work) following alcohol consumption at home. During the confrontation, the victim grabbed the perpetrator around the neck, prompting the latter to struggle and retaliate by striking the victim on the chest with his elbow. Immediately, the victim collapsed to the ground in apparent pain. Approximately 2 min later, the victim lost consciousness. The perpetrator, believing the victim to be intoxicated, moved him to a bed. Approximately 30 min later, the victim was found not breathing. An older female relative, lacking first aid training, performed cardiopulmonary resuscitation (CPR) for 2 min and called for emergency services. The ambulance arrived 15 min later and immediately performed CPR, but the victim was pronounced dead after 20 min of advanced resuscitation attempts.

**Figure 1:**
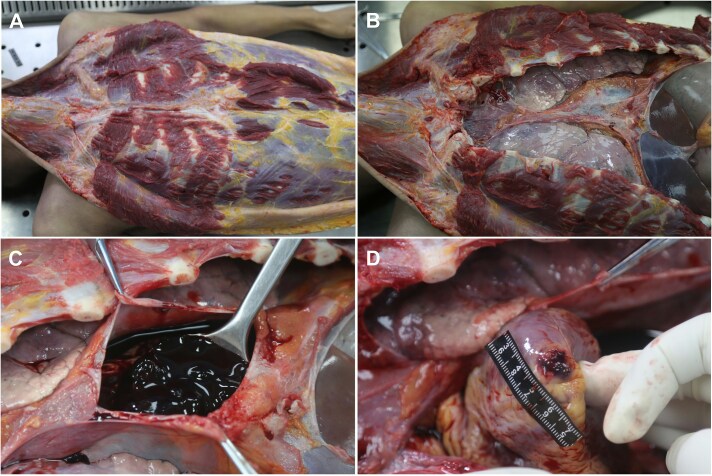
Macroscopic findings of the chest wall and cardiac rupture. (A) The chest wall shows no soft tissue injuries or fractures. (B) Intact pericardium. (C) Blood and clots within the pericardial cavity. (D) A 0.3 cm × 0.1 cm rupture at the left ventricular apex.

**Figure 2:**
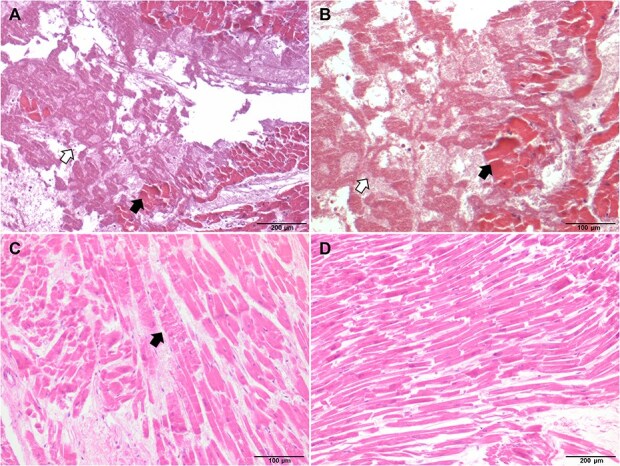
Microscopic observation of the myocardial rupture site. (A) Coagulation necrosis of cardiomyocytes (black arrow), haemorrhage, fibrin, and platelet thrombus (white arrow) around the cardiac rupture site (100×). (B) A higher-magnification view of the area in (A) (200×). (C) Contraction band necrosis in cardiomyocytes adjacent to the rupture site (black arrow, 200×). (D) Normal myocardial tissue from the remaining left ventricular wall (100×).

The body was stored at −20°C until the autopsy was performed 2 days later. The victim measured 176 cm in height. External examination revealed several minor bruises on the left side of his face and left elbow. There were no injuries to the chest wall, sternum, ribs, or pericardium (Figure 1A and B). The pericardium was intact; however, 300 mL blood and 100 g of clots were found in the pericardial cavity (Figure 1C). An oval rupture measuring 0.3 cm × 0.1 cm was found on the left ventricular apex (Figure 1D). The heart was normal in size and weighed 240 g. No abnormalities were observed in the ventricular cavity or coronary artery. The thicknesses of the left ventricular wall and right ventricular wall were 1.3 and 0.4 cm, respectively. Coagulation necrosis and contraction band necrosis of the cardiomyocytes, haemorrhage, fibrin, and platelet thrombus were observed around the rupture (Figure 2A–C). The remaining portion of the myocardium was unremarkable (Figure 2D). The sinoatrial and atrioventricular nodes and his bundle showed no abnormalities. A contusion measuring 7.0 cm × 5.0 cm was observed at the root of the right lung. No significant pathological findings were noted on other organs. The ethanol concentration in cardiac blood was 0.213 g/dL. Other common toxin screening tests, including those for common organophosphorus pesticides and sedative drugs, were negative in cardiac blood and gastric contents. Medical and family histories of the victim were unremarkable. The victim had no history of drug abuse, and the cause of death was attributed to acute cardiac tamponade caused by blunt cardiac rupture.

## Discussion

In forensic practice, cardiac ruptures similar to the one presented here can be diagnostically challenging. Firstly, the possibility that CPR caused the rupture must be considered and ruled out. While CPR involves blunt chest trauma, it is an uncommon cause of blunt cardiac rupture [7–10]. CPR-related cardiac ruptures share features with traumatic ones, including the rupture site and the potential associated injuries [11–13]. However, CPR is often performed during the agonal period or after death. Consequently, haemorrhage from CPR-related cardiac ruptures is usually much less extensive than that from ante-mortem blunt trauma. Furthermore, the thrombi around the rupture and clots in the pericardial cavity are unlikely to occur after death [14]. In this case, the aforementioned pathological evidence indicates that the cardiac rupture occurred before death. Moreover, in the present case, the two rounds of CPR were conducted about 30 and 45 min, respectively, after the elbow strike. The first CPR attempt was performed by an untrained individual for only 2 min when the victim was found not breathing. No vital signs were recorded during the second CPR cycle. Finally, cases of CPR-induced rupture often present with another explicable cause of death [7], which was not observed here. Therefore, CPR was ruled out as the cause of the cardiac rupture. Nevertheless, we should be aware of the occasional deaths caused by CPR-induced cardiac ruptures, which may occur when individuals receive prolonged and non-professional CPR [7].

In this case, the victim’s presentation at the time of the strike was consistent with the typical symptoms of cardiac rupture and pericardial tamponade. Cardiac rupture of the left ventricular free wall with an intact pericardium usually leads to rapid death [15]. Given the close temporal sequence of the victim’s collapse immediately after the injury, the evidence strongly suggests that the cardiac rupture was caused by the strike.

Secondly, the presence of any myocardial pathology that could cause cardiac rupture must be determined. Cardiac rupture is a fatal complication of acute myocardial infarction [16]. Older age, presence of acute or old myocardial infarction, and severe coronary artery occlusion are common features of cardiac rupture caused by myocardial infarction [17]. In this case, the victim had no atherosclerotic plaques in the coronary arteries. Although coagulation necrosis and contraction band necrosis of cardiomyocytes were observed around the rupture site, they were interpreted as secondary phenomena after cardiac rupture. Thus, cardiac rupture secondary to myocardial infarction was excluded.

Thirdly, Takotsubo cardiomyopathy (TCM) was included in the differential diagnosis. TCM is characterized by acute, transient balloon-like dilation of the left ventricular apex without coronary atherosclerosis. Furthermore, cardiac rupture of the apex is a rare but severe complication of TCM [18, 19]. However, TCM is more prevalent in older postmenopausal women and is often triggered by emotional or physical stressors [20]. Myocardial biopsy in TCM typically reveals areas of necrosis, inflammatory cell infiltration, and localized fibrosis [18, 21]. This myocardial fibrosis reduces the compliance of the ventricular free wall, which may lead to apex rupture during the abnormal dilation in TCM [18]. In this case, the victim, a young man, had no history of stressful events or cardiovascular disease. In contrast, the unaffected myocardium (apart from the rupture site) showed no interstitial fibrosis or inflammatory cell infiltration. Moreover, as myocardial fibrosis is a chronic condition, patients with TCM may experience prolonged cardiac discomfort prior to cardiac rupture [22]. The instantaneous death in this case is not characteristic of TCM, although the precise clinical course of TCM is not explicit. Therefore, TCM was ruled out as the cause of cardiac rupture in this young man.

In this case, no other potential cause of death was found. The contusion at the root of the right lung indicated blunt trauma. Although the literature shows that blunt cardiac injuries are more likely to be accompanied by external and chest injuries [2, 6, 23], understanding left ventricular rupture without any external chest injuries in an adult, as in this case, is challenging. However, a diagnosis of blunt cardiac rupture was established after excluding other potential causes.

Many factors influence the severity and extent of cardiac injuries. The sudden enormous pressure applied to the chest is a critical factor in blunt cardiac rupture [24]. Male athletes can deliver a force greater than 5.1 kN [25] within a small contact area [26], which is sufficient to cause head injuries [25], duodenal stump perforations [27], and spleen injuries [28]. In this case, the perpetrator was a young adult male engaged in strenuous physical labour. Moreover, he struggled to free himself from the victim’s strangling. Therefore, the elbow strike sustained by the victim may have been considerably forceful. Other factors, including the timing of force application during the cardiac cycle and the compliance of the chest wall, may also contribute to blunt cardiac rupture [29–31]. Cardiac ruptures caused by blunt trauma typically occur at the end of diastole [14]. At this point, the ventricular cavities are filled with blood, and their walls are thinner, rendering them more vulnerable to external force [14]. Typically, the left ventricular apex is the thinnest part of the left ventricular free wall [32]. Thus, the cardiac apex may be the most vulnerable site during this phase. In this case, cardiac the rupture occurred precisely at the left ventricular apex.

Chest wall compliance is another influencing factor in blunt traumatic cardiac ruptures. Greater chest wall compliance indicates easier force transmission. Severe cardiac injuries without external injuries can occur in children, primarily because of the high elasticity and flexibility of their chest walls [29–31, 33, 34]. The absence of external injury in this case can be explained by the high pliability of the victim’s chest wall, even though he was 21 years old. The absence of rib or sternal fractures from CPR further supports this interpretation. Thus, it is possible for the young man to experience significant focally inflicted internal injury without accompanying external injury.

In addition, ethanol intoxication cannot be ignored in this case. Some studies indicate that even moderate alcohol consumption can compromise the stability, coordination, reactivity, and motor and perception functions of the body [35]. Turkmen et al. [34] reported a sleeping girl who died of cardiac rupture following a fall, in which the mechanical compromise in sleeping might have be similar to the intoxicated situations in this case.

## Conclusion

This case reminds forensic pathologists that cardiac rupture can be caused by blunt trauma in young people even in the absence of thoracic bone fractures or visible external injuries. The cause of cardiac rupture is difficult to determine in forensic practice because of miscellaneous situations. To diagnose blunt traumatic cardiac ruptures, CPR-related cardiac ruptures must first be excluded. Pre-existing myocardial pathology and TCM should be carefully evaluated. A definitive diagnosis of blunt traumatic cardiac rupture can be established only after excluding these alternative causes. Certainly, it is crucial to ensure that no explicable cause of death other than cardiac rupture in cases of blunt cardiac rupture. Many factors, such as sudden enormous force, cardiac cycle, chest wall compliance, and alcohol consumption, likely contributed to the cardiac rupture in this case. It is the foundation for delineating legal responsibility in judicial practice to accurately distinguish between cardiac ruptures caused by trauma, underlying disease, or medical intervention.

## Data Availability

Not applicable.
